# Genome analysis of a cluster EF bacteriophage LordBart isolated from soil in Tennessee.

**DOI:** 10.17912/micropub.biology.001929

**Published:** 2026-01-15

**Authors:** Sergei Markov, Cynthia Fecteau, Torrie Jones, Matthew Lee, Mercedes Thornton

**Affiliations:** 1 Biology, Austin Peay State University, Clarksville, Tennessee, United States

## Abstract

Bacteriophage LordBart was isolated from a soil sample in Clarksville, TN using the bacterium *Microbacterium foliorum.* The bacteriophage has a 56,975 bp genome with 86 predicted protein-coding genes, of which 32 were assigned predicted functions. LordBart has a siphovirus morphology and is grouped with bacteriophages in cluster EF based on gene content similarity. Its genome includes eight copies of a conserved 12 bp sequence motif located upstream of predicted translational start codons of some genes of unknown functions.

**Figure 1. Transmission electron micrograph of bacteriophage LordBart displaying a siphovirus morphology with an icosahedral capsid of diameter 52 - 54 nM and a 132 - 134 nM long flexible tail (n=6):**
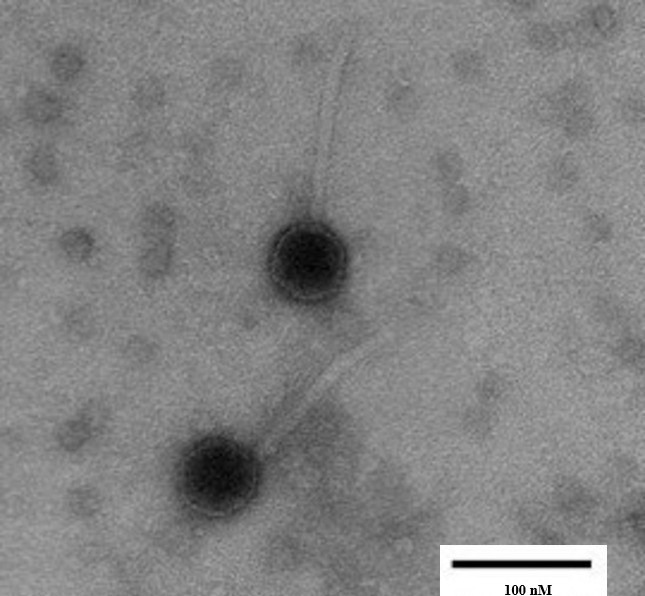
Bacteriophage samples were stained using 1% uranyl acetate on grids attached to Pelco Tabs (Ted Peller, Inc., Redding, CA). A Hitachi H-7650 Transmission Electron Microscope (Tokyo, Japan) with an accelerating voltage of 100 kV was used to generate bacteriophage images.

## Description

Bacteriophages play important roles in regulating the population of bacteria and have applications in biotechnology and medicine (Jacobs-Sera et al., 2020; Hatfull, 2020; Markov et al. 2024).&nbsp; To broaden our understanding of actinobacteriophage diversity and evolution, we describe the isolation and characterization of bacteriophage LordBart.

The bacteriophage was isolated from moist soil in Clarksville, Tennessee (GPS coordinates 36.608459 N, 87.390453 W) using *Mycobacterium foliorum* NRRL B-24224 as the host and by following standard methods (Russell et al., 2019; Zorawik et al., 2024).&nbsp; The soil sample was collected when the ambient temperature was 19°C and then suspended in peptone-yeast calcium (PYCa) liquid medium for 2 hours.&nbsp; Subsequently, the suspension was passed through a 0.22- µM-pore filter, and the filtrate was inoculated with *M. foliorum* and incubated with shaking at 250 rpm for 2 days at 30°C.&nbsp; The culture was then filtered again and the filtrate (10 µL) plated in PYCa top agar with *M. foliorum* and incubated for 2 days at 30°C.&nbsp; After incubation, a plaque was selected and purified through two additional rounds of plating, yielding phage LordBart, which formed clear, round plaques.&nbsp; Transmission electron micrograph of LordBart showed that it has the siphoviral morphology (Fig. 1).&nbsp;

DNA was isolated from a lysate of LordBart using the Wizard DNA Clean-Up Kit (Promega, Madison, WI).&nbsp; It was enzymatically sheared for sequencing using the Ultra II Library Kit (NEB, Ipswich, MA). &nbsp;An Illumina NextSeq 1000 with an XLEAP-P1 Kit was used for DNA sequencing to yield 100-base single-end reads with 2423-fold coverage.&nbsp; Raw reads were trimmed with cutadapt 4.7 (using the option: –nextseq-trim 30), filtered with skewer 0.2.2 (using the options: -q 20 -Q 30 -n -l 50), then assembled using Newbler v2.9 (Russell, 2018). &nbsp;Raw reads were checked for genomic termini and completeness using Consed v29 (Gordon et al., 1998) as previously described by Russell (2018). &nbsp;The LordBart has a circularly permuted genome DNA of 56,975 bp size with GC content of 62.7%.&nbsp;

Lord Bart’s genome was automatically annotated in DNA Master v5.23 (Pope et al *.,* 2018) using Glimmer v3.02 (Delcher et al., 1999) and GeneMark v2.5p (Besemer and Borodovsky 2005). The annotated genes calls were then manually refined using using Starterator v485.0 ( http://phages.wustl.edu/starterator ) while putative functions were assigned using Phamerator v393.0 (Cresawn et al., 2011), PECAAN v20211202.0 ( https://blog.kbrinsgd.org/ ), BLASTp (Altschul et al., 1990) against the NCBI non-redundant and Actinobacteriophage databases, HHpred v3.2 (Söding et al., 2005) against the PDB_mmCIF70, Pfam v.37.0, and NCBI Conserved Domains databases v3.19. &nbsp;Default parameters were used for all programs. &nbsp;As a result of this annotation process, 86 protein-coding genes were predicted in LordBart, of which putative functions were assigned for 32 genes.&nbsp; Using the gene content similarity of at least 35% to bacteriophages in the Actinobacteriophage database, LordBart was placed in actinobacteriophage cluster EF (Pope et al., 2017; Gauthier and Hatfull 2023).

The LordBart genome contains eight copies of a conserved 12 bp sequence motif 5’-GGGAAGGAACCC before genes 15, 16, 41, 52, 53, 67, 64 and 68, contrary to a previously reported 13 bp motif (5’-GGGAAAGGACCCC) for other EF cluster bacteriophages, AnnaSerena and Krampus (Jacobs-Sera et al., 2020).&nbsp; LordBart has this 13 bp motif only before gene 64.&nbsp; The motif is located directly (or a couple of nucleotides away) upstream of predicted translational start codons, such as ATG.&nbsp; This motif may thus be important for regulating bacteriophage gene expression and may also be involved in genome replication.&nbsp; To date, none of the genes directly preceding this motif have known functions.&nbsp;

&nbsp;

**Data availability**

GenBank accession number for LordBart is PV915886 and SRA accession number is SRX28150556.
